# Renal Involvement in Antiphospholipid Syndrome

**DOI:** 10.3389/fimmu.2018.01008

**Published:** 2018-05-17

**Authors:** Alonso Turrent-Carriles, Juan Pablo Herrera-Félix, Mary-Carmen Amigo

**Affiliations:** ^1^Internal Medicine Rheumatology Service, Centro Médico ABC, Mexico City, Mexico; ^2^Nephrology Department, Centro Medico ABC, Mexico City, Mexico

**Keywords:** antiphospholipid syndrome, systemic lupus erythematosus, renal disease in antiphospholipid antibody syndrome, antiphospholipid antibody syndrome nephropathy, renal thrombotic microangiopathy

## Abstract

Antiphospholipid syndrome is a complex autoimmune disease, characterized by the presence of vascular thrombosis, obstetric, hematologic, cutaneous, and cardiac manifestations. Renal disease in patients with antiphospholipid syndrome was not recognized in the first descriptions of the disease, but later on, the renal manifestations of the syndrome have been investigated widely. Renal manifestations of antiphospholipid syndrome conform a wide spectrum of diverse renal syndromes. Hypertension is one of the most frequent, but less commonly recognized renal alteration. It can be difficult to control as its origin is renovascular. Renal vascular thrombosis can be arterial or venous. Other alterations are renal infarction and vascular thrombosis in arterial territories. Venous thrombosis can be present in primary and secondary antiphospholipid syndrome; it presents with worsening of previous proteinuria or *de novo* nephrotic syndrome, hypertension and renal failure. Antiphospholipid syndrome nephropathy is a vascular disease that affects glomerular tuft, interstitial vessels, and peritubular vessels; histopathology characterizes the renal lesions as acute or chronic, the classic finding is thrombotic microangiopathy, that leads to fibrosis, tubule thyroidization, focal cortical atrophy, and glomerular sclerosis. Antiphospholipid syndrome nephropathy can also complicate patients with systemic lupus erythematosus, and there is vast information supporting the worse renal prognosis in this group of patients with the classic histopathologic lesions. Treatment consists of anticoagulation, as for other thrombotic manifestations of antiphospholipid syndrome. There is some evidence of glomerulonephritis as an isolated lesion in patients with antiphospholipid syndrome. The most frequently reported glomerulonephritis is membranous; with some reports suggesting that immunosuppressive treatment may be effective. Patients with end stage renal disease commonly are positive for antiphospholipid antibodies, but it is not clear what is the role of aPL in this setting. Patients with vascular access may have complications in the presence of antibodies so that anticoagulation is recommended. Patients ongoing renal transplant with persistent antiphospholipid antibody positivity may have early and late graft failure.

## Introduction

Antiphospholipid antibody syndrome (APS) is a complex autoimmune systemic disease, characterized by the presence of circulating antibodies directed against anionic phospholipids, and the proteins bound to them (aPL) in the serum of patients with thrombosis or pregnancy complications. There are classic manifestations of APS, including thrombosis involving arterial and venous territories and obstetric morbidity, that are considered as classification criteria (1). Moreover, there are many other manifestations of APS, the “non-criteria” manifestations that include livedo reticularis, hematologic manifestations (thrombocytopenia and hemolytic anemia), cardiac valve disease, and renal involvement.

Renal involvement was not mentioned in the first description of APS (2). Kidney compromise in APS represents a vast and complex myriad of syndromes that are a consequence of the vascular dysfunction and the coagulation dysregulation characteristic of the syndrome. Kidney disease associates with aPL is not an inflammatory condition in contrast with lupus nephritis. Recently, many groups are interested in this frequent complication of APS (3–5).

All the vessels, veins, and arteries, from the renal arteries to the glomerular tuft capillaries can be involved. Table 1 shows the renal syndromes that are related to APS.

**Table 1:** Renal involvement in antiphospholipid antibody syndrome (APS).

a) Hypertension b) Renal artery stenosis, thrombosis, and infarction c) Renal vein thrombosis d) Intrarenal vasculopathy [APS nephropathy (APSN)] e) Glomerular disease f) APS in kidney transplant g) APS in end stage renal disease and hemodialysis h) APSN in patients with systemic lupus erythematosus i) APSN in catastrophic APS

The real prevalence of renal involvement in APS is very difficult to establish, mainly due to the limitation of histopathology research, biopsy contraindications, and its association with lupus (SLE). Retrospective series have mentioned a prevalence of 9–10% (6), but in series where APS renal disease has been intentionally studied the prevalence ranges from 10–40% (7–9).

### Hypertension

Hypertension is a fairly common health problem in the adult population. Depending on the definitions used for classifying patients with high blood pressure (JNC8 or ACC/AHA 2017), 32–46% of adults has hypertension (7, 8). According to the last ACC/AHA definitions, a normal blood pressure is <120/<80 mmHg, elevated blood pressure 120–129/<80 mmHg, stage 1 hypertension 130–139/80–89 mmHg, and stage 2 hypertension >140/>90 mmHg (10, 11).

Since the initial descriptions of APS, hypertension was one of the frequent signs related to the disease. Hughes in 1983, described patients with livedo reticularis in association with elevated blood pressure, suggesting a renovascular etiology. In 1986, he described a group of patients with APS and hypertension, which ranged from mild elevation to malignant hypertension (2, 12).

The etiology of the elevated blood pressure within this group of patients is thought to be renovascular in origin, since there are case reports (13, 14), and series where intrarenal vascular lesions demonstrated in biopsies, were the only physiopathologic explanation. In a large series of renal biopsies in patients with APS, Nochy et al. found systemic hypertension on 93% of their patients, this being the most common clinical manifestation of APS nephropathy (APSN). Hypertension was severe in 31% of the patients and malignant in 12% (15). Taking into account the high prevalence of hypertension in APSN, elevation of blood pressure is considered one of the most important signs that suggest renal activity.

Some studies have suggested that the presence of aPL is related directly to hypertension. Rollino et al. compared healthy controls with matched hypertensive patients and patients with renal artery stenosis, finding that 8% of the patients with essential hypertension had aPL vs. none of the healthy controls (16). Frostegard et al. analyzed the presence of anti-B2GP1 in patients with hypertension vs. matched controls. They found that the presence of IgG aPL correlated with elevated levels of insulin, insulin-like growth factor binding protein-1, and more insulin resistance, suggesting that patients with anti-B2GP1 have more or are prone to develop more atherosclerotic lesions and higher blood pressure (17).

Hypertension in patients with APS may be very difficult to treat, taking into account that its exact nature is only partially understood. Nowadays, the best treatment for these patients is anticoagulant therapy and optimal blood pressure control with antihypertensive drugs. With this combination, the progression to end stage renal disease (ESRD) could be delayed or even prevented (18). Treatment with prednisone has also anecdotically been reported with good results.

### Renal Artery Thrombosis, Stenosis, and Renal Infarction

Renal artery disease is an infrequent but well recognized manifestation with important consequences in APS patients. Since 1990 when Ostuni et al. reported for the first time the occurrence of renal artery thrombosis and hypertension in a young patient with anti-phospholipid antibodies (19), many similar cases have been published (20–23). Renal artery disease can present as renal infarction, ischemic acute renal failure, slowly progressive chronic renal failure, or renovascular disease. The most common clinical picture in this group of patients is the new onset of severe hypertension or given the case worsening of a previously documented and controlled hypertension. Other clinical features are lumbar pain, localized around the renal area, hematuria, or renal failure.

Sangle et al., in an elegant study, reported two different patterns of arterial disease in APS patients. With magnetic resonance angiography, they visualized the renal arteries of 77 APS patients with resistant hypertension and compared them with the arteries of young hypertensive patients and healthy controls. In the APS group, 20 (28%) of the patients had renal artery lesions, compared with 8% in young hypertensive patients and 3% of healthy controls. Moreover, they reported two different kinds of renal artery lesions. The first and more common one was characterized by a smooth, well delineated, non-critical stenosis localized distal to the ostium of the renal artery. The second was similar to the common atherosclerotic lesions presented in other metabolic and chronic diseases. They were located proximal to the ostium and may involve the aorta (24). In APS, there is accelerated atherosclerosis and could be associated with renal lesions (25). Vasoconstrictive mechanisms observed in APS are related to high endothelin-1 levels (26).

Different imaging studies have been useful to visualize the arterial alterations, including renal ultrasound with Doppler, abdominal CT, gadolinium magnetic resonance angiography, renal angiography, and renal scintigraphy have proved their usefulness. Ultrasound may be the first screening method, followed by CT or magnetic resonance. An algorithm approach has been proposed (27).

In patients presenting with APS, renal artery disease, and hypertension, treatment based on anticoagulant therapy requires a concomitant strict blood pressure control. On a retrospective study, Sangle et al. analyzed blood pressure response of patients receiving anticoagulant therapy. Patients with higher INR (>3.0) had better blood pressure control, maintained renal function, and arterial lesions reversed in some patients (28). Blood pressure and renal function maintenance during anticoagulant therapy suggest a thrombotic etiology for the arterial lesions presented in most patients with APS. Some studies have demonstrated successful thrombolysis and balloon angioplasty; however, anticoagulation was used in all patients.

Renal infarction, another manifestation of the APS is caused either by *in situ* thrombosis affecting renal arteries, infrarenal aorta, smaller diameter intraparenchymal vessels, or due to embolic disease from a pre-existing upstream arterial lesion or altered cardiac valves (29–31).

Renal infarcts are characterized clinically by intense lumbar pain that may be unilateral or bilateral, accompanied by hypertension and acute renal injury. Renal infarction may be the initial clinical manifestation of APS (32, 33).

The histological features are glomerular ischemia, tubular atrophy, and interstitial fibrosis. Histological changes consistent with thrombotic microangiopathy (TMA) are not present in this subgroup (29, 34).

Subclinical cases have also been reported as an incidental finding when on CT scans performed with other purpose, images revealed an old silent infarct. It is suggested to perform anti-phospholipid testing only in young patients with no other identified cause of subclinical renal infarction.

Patients with SLE that are stable with treatment, mainly hydroxychloroquine, at the moment of pharmacologic withdrawal may have this complication (35).

### Renal Vein Thrombosis

Patients with primary APS or more frequently patients with SLE and APS may present renal vein thrombosis. Thrombosis may involve the inferior vena cava or the main and minor renal veins.

Asherson et al. published the first reported cases, describing two patients with SLE and proliferative nephritis with nephrotic syndrome and positive lupus anticoagulant (36). Glueck et al. reported three cases of renal vein thrombosis in patients with SLE and positive lupus anticoagulant (37). These studies associated renal vein thrombosis with the presence of lupus anticoagulant.

The most common clinical manifestation of renal vein thrombosis is nephrotic range proteinuria, and occasionally, renal failure when thrombosis is bilateral.

Sudden onset or acute worsening of nephrotic range proteinuria should make the clinician suspect this complication. Doppler of renal vasculature enhanced CT or MRI are the studies recommended to confirm or rule out this complication (31, 38, 39). Other causes of renal vein thrombosis like pregnancy, oral contraceptive use, extrinsic compression, trauma, and other nephrotic syndrome causes, should also be evaluated (30).

### Intrarenal Vascular Lesions: APSN

The APSN is considered a vascular nephropathy that can present acutely or chronically. Patients with primary and secondary APS have shown the classic histopathologic lesions of APSN.

D’Agati et al. published the first reports in 1990, who described three patients, two with primary APS and one with SLE who had acute TMA on renal biopsy (40). Becquemont and coworkers reported one case of renal microangiopathy associated with anti-cardiolipin antibodies (41).

**Figure 1:**
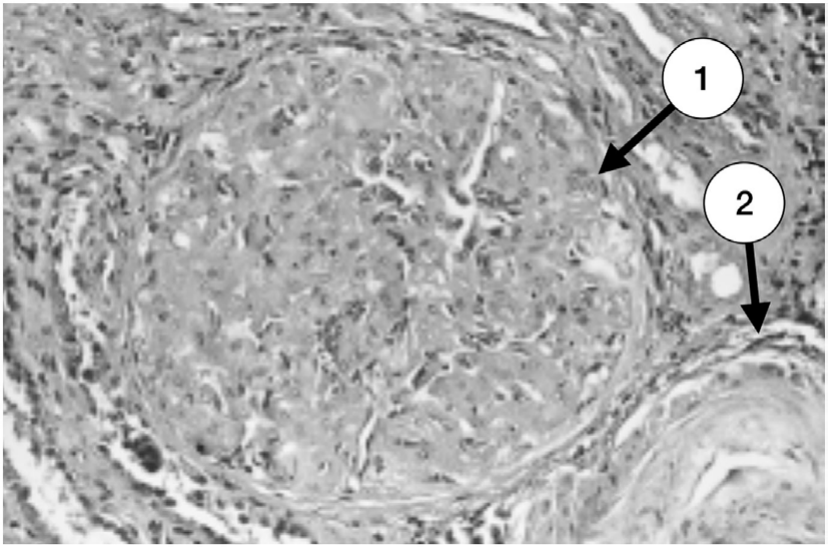
Glomerulus with severe and advanced thrombotic microangiopathy. Capillary lumina are occluded by mesangiolysis and heterogeneous subendothelial deposits (1). An arteriole with a great deal of lucent subendothelial deposits is partially seen at the right inferior corner (2) (with permission of the publisher).

**Figure 2:**
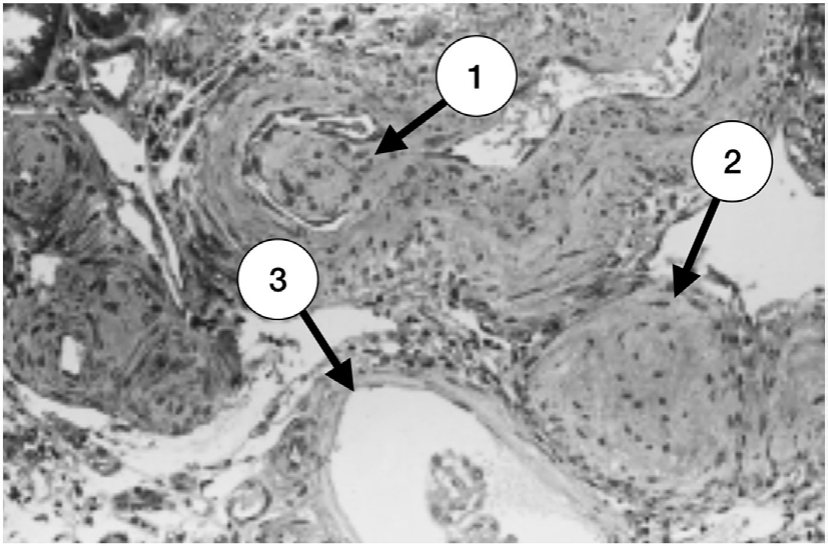
Late stage of thrombotic microangiopathy in a small arteriole. There is fibrotic medial hyperplasia and the lumen is irregular. A fibrotic intraluminal “cushion” caused by a mural thrombus organization is shown in (1). There are also fibrohyperplastic arterioles and two glomeruli; one is ischemic (2) and the other, fibrotic (3) (with permission of the publisher).

**Figure 3:**
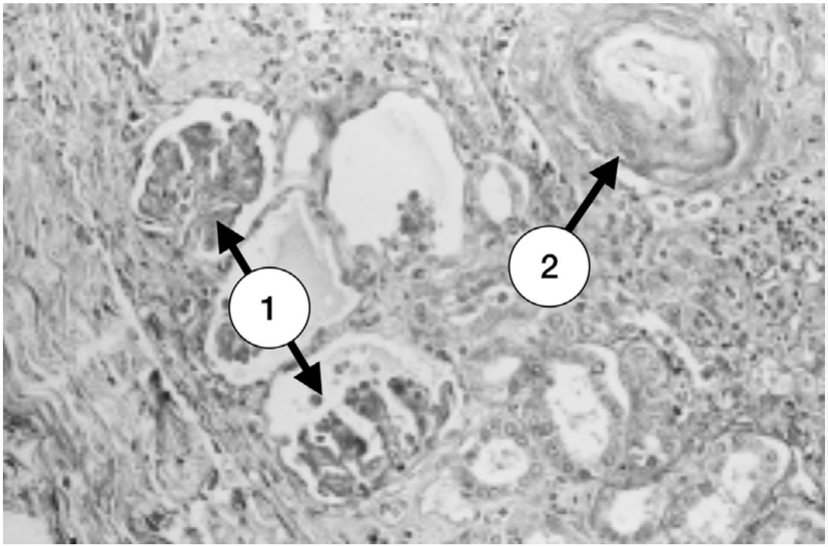
A small focus of superficial ischemic cortical atrophy. There are several ischemic glomeruli with tuft retraction and Bowman’s space enlargement (1). At the right upper corner a small vessel with a great amount of a subendothelial clear material and marked narrowing of the lumen is seen (2) (with permission of the publisher).

Amigo et al. described the correlation between the clinical characteristics and the pathologic findings in five patients with primary APS and renal involvement. All the patients had hypertension, three had mild renal impairment, and two had ESRD requiring renal substitution therapy (hemodialysis). Renal biopsies were consistent with TMA, with acute and chronic vascular lesions (Figures 1–3). Subendothelial fibrosis and arteriolar luminal narrowing were also found (42).

In 1999 Nochy et al. studied retrospectively 16 patients with primary APS and renal involvement, all of them had a previous renal biopsy (15). The following histologic lesions are the ones described and supported the actual definition of APSN:

– Arteriosclerosis characterized by fibrous intimal thickening with luminal reduction of arcuate and interlobular arteries, associated with arteriolar hyaline and arteriolosclerosis.
– Fibrous intimal hyperplasia (FIH) whose characteristics are thickened intima and intense myofibroblastic intimal cellular proliferation in interlobular arteries and their branches. The media shows proliferative changes with hypertrophic myocytes or atrophic and fibrous changes.
– Fibrocellular and arteriolar occlusion in small diameter interstitial arteries.
– TMA that commonly affects preglomerular arterioles, small interlobular arteries, and glomerular capillaries. The histologic pattern is non-inflammatory with occlusion of vessel lumen by red blood cell fragments, leukocytes, and eosinophilic fibrinoid material. When analyzed by immunofluorescence fibrin is the only material of the thrombi and immunoglobulins are absent.
– Vasculitis is typically absent.
– Focal cortical atrophy (FCA) involves superficial cortex under the renal capsule, disposed of as foci or triangles, with depression of the contour of the renal capsule. All of the elements of the renal parenchyma can be affected, creating lesions that are typical of APSN. The glomeruli can appear sclerotic or pseudocystic and voluminous. The immunofluorescence reveals fibrin and sometimes C3 and IgM deposits in the vessels showing thickened cellular intima. Renin can be found in the juxtaglomerular apparatus and the wall of the interlobular arteries.
– Tubular thyroidization is characterized by zones with tubular atrophy containing eosinophilic casts that resemble thyroid tissue. These zones are frequently found in the deep cortex or medulla.

Classically, acute vascular lesions are secondary to TMA, and the other histologic patterns are chronic. The typical histological features of APSN are the combination of TMA with chronic lesions. Other ultrastructural changes are typical of APSN including mainly glomerular basement membrane wrinkling and reduplication (42, 43).

It is important to recognize that none of the histological patterns is pathognomonic of APSN, since the lesions can be present in malignant hypertension, scleroderma renal crisis, HIV, cyclosporine use, chemotherapy, preeclampsia, and thrombotic thrombocytopenic purpura/hemolytic uremic syndrome (TTP/HUS) (7, 44). The distinction between APSN and TTP/HUS can be made by the presence of schistocytes on blood smear, severe thrombocytopenia, negative aPL, and lack of microcirculatory thrombosis, which are characteristics of TTP/HUS and not of APSN (45).

The clinical features of APSN are hypertension (generally severe), acute or chronic renal injury, proteinuria (mild to nephrotic), and hematuria (31).

Treatment of APSN includes antihypertensive agents aiming strict control of blood pressure, oral anticoagulation with vitamin K inhibitors, and there are some small series addressing the benefit of immunosuppressive therapy in APSN (46, 47). Korkmaz et al. reported benefit treating patients with steroids, azathioprine, and cyclophosphamide with good response (46). A phase II trial with rituximab (RITAPS) showed efficacy in two cases with APSN (48), but further information regarding this topic is needed. The use of C5a inhibition with eculizumab may be an option in patients with TMA, but more information is needed to support its recommendation.

The prognosis of APSN is variable with a high prevalence of chronic hypertension reported in most series. Proteinuria, nephrotic syndrome, chronic renal failure, or ESRD may also occur.

Catastrophic APS (CAPS) is a very rare (<1%) and extremely severe variant of APS. It is characterized by multiple systems and thrombotic organ involvement that occurs in a very short period (days to weeks). Renal involvement is a common feature in CAPS, the most frequent finding is TMA, but other chronic lesions of APSN can also be found (49). The treatment of CAPS includes high dose steroids, anticoagulation, IV Immunoglobulin, and plasma exchange. In patients with CAPS associated with SLE, cyclophosphamide may be effective. Moreover, eculizumab has been succesfully used in few cases.

### Glomerular Involvement in APS

Besides the classic APSN that typically consists of vasculopathy and intrarenal thrombosis, there is enough evidence that other clinical and histopathologic patterns can be present in patients with APS.

Fakhouri et al. in 2003 retrospectively studied the pathologic patterns of 29 renal biopsies of patients with primary APS and no evidence of another autoimmune disease. In this study, 20 biopsies had findings characteristic of classic APSN, and the other nine biopsies had different patterns that included three membranous glomerulonephritis, two with mesangial C3 nephropathy, two with minimal change disease, one with focal segmental glomerulosclerosis, and one biopsy had mixed changes consistent with pauci-immune vasculitis and focal segmental glomerulosclerosis. Seven cases had a subacute or chronic clinical course, and two of them presented acute renal failure. All cases had relevant proteinuria and five patients presented nephrotic syndrome (50).

Sinico et al., studied retrospectively 160 APS patients demonstrating renal involvement in 14 (8.7%) patients. Ten patients underwent renal biopsy, four of them had membranous glomerulonephritis, two had diffuse proliferative glomerulonephritis, and the other four had classic pathologic findings consistent with APSN. Patients with membranous glomerulonephritis had lower levels of complement. None of the patients developed SLE on follow up (6).

Membranous glomerulonephritis is the most frequently reported glomerular disease in APS in different series and case reports (6, 37, 40, 50–55). Quereda et al. analyzed the frequency of aPL in different non SLE nephropathies, finding aPL in 20% of the patients with membranous nephropathy, 2 of them fulfilling classification criteria for APS (56).

Even though glomerulonephritis is infrequent in patients with APS, there is enough evidence and information that this kind of renal disease is related with APS, and they should be taken in account when analyzing renal biopsies from patients with APS. There are no studies regarding the treatment in this group of patients, probably the best treatment is a combination of immunosuppressive drugs with anticoagulation, but studies are needed to support the recommendation.

### APSN in Patients With SLE

Patients with SLE can have persistent positivity to aPL, with a prevalence of 15–60% depending on the series. However, only 30% of them have APS. Patients with aPL in SLE commonly have a history of thrombosis, obstetric morbidity, and hematologic alterations.

Considering APSN as a renal dysfunction caused primarily by capillary thrombosis, FIH, FCA, or TMA, Kant and Glueck reported higher glomerular capillary thrombotic lesions initially in SLE patients with positive aPL compared with patients with negative aPL (37, 57). The prevalence of APSN in SLE varies between 11% to more than 50%, but most series are retrospective, and the pathologists used different criteria to define the presence of APSN on SLE renal biopsies (7, 9, 58, 59).

Vascular thrombotic lesions that are typical of APSN can be isolated or associated with the classic lesions of lupus nephritis. The clinical manifestations in patients with APSN in SLE are hypertension, nephrotic syndrome, and renal dysfunction.

Most studies have reported poor renal prognosis in patients with SLE and coexisting APSN. However, Naiker et al. reported a high prevalence of aPL in patients with SLE nephritis but did not found worse renal prognosis (60).

One of the most relevant studies addressing prognosis, analyzed prospectively 111 patients with SLE nephritis followed for 14 years. 26% of the patients were aPL positive, and those patients had a poor renal outcome, higher creatinine levels, and higher chronicity index on biopsy (52).

Bhandari et al., in a cohort study, found a relevant association of positive aPL and a higher prevalence of crescentic, sclerotic, and glomerular necrosis in renal biopsies of SLE patients, supporting the worse prognosis conferred by aPL (61).

Tektonidou et al. studied the natural history of APSN performing repeated renal biopsies. They found the progression from acute capillary thrombosis to chronic obstructive and fibrotic lesions. TMA was followed by chronic lesions, such as FIH, FCA, or sclerotic lesions. Since the evolution to chronic lesions conferred a worse prognosis, it is extremely important to recognize the acute histologic findings in an early period (7).

A recent study by Barrera-Vargas et al. compared renal function outcome between SLE patients with TMA associated with lupus nephritis and patients with isolated lupus nephritis. The authors did not find an association with positive aPL. However, patients with TMA had worse renal prognosis (62).

As patients with SLE and APSN tend to have a worse prognosis, it is crucial to document the presence of APSN in a kidney biopsy. A renal biopsy must be done with great caution because these patients have an increased risk of bleeding after the procedure (63). When SLE nephritis predominates, immunosuppressive therapy with mycophenolate or cyclophosphamide must be used, and when APSN is found, anticoagulant therapy must be added.

### ESRD and Renal Transplantation

Progression to ESRD is an uncommon course in patients with APS. Erkan et al. in a prospective study that included 39 patients with APS found that only 1 patient developed ESRD during the 10 years follow up (64). Other studies have investigated this relationship (35). Sinico et al., studied retrospectively 160 APS patients, and only 1 developed ESRD (6). Amigo et al. studied 20 consecutive primary APS patients finding acute and chronic TMA lesions in renal biopsies of 5 patients. Two of these five patients presented ESRD (42). This poor renal outcome is uncommon in children with APS (65).

Patients with ESRD independently of its cause have a higher frequency of aPL positivity compared with the general population. Different studies have assessed these findings (18, 66–71), but the patient characteristics and antibody assays were different in each study.

The proposed mechanisms to explain aPL positivity in patients with ESRD are: uremia as an altered immunogenic state predisposing to autoimmunity (72), antibody induction by dialysis membrane incompatibility (71), blood trauma generated in hemodialysis circuits (73), and, induction by microbial products like endotoxins present in dialyzate (71). However, there is no explanation why only a few patients using the same membranes, different length of time on dialysis or using the same dialyzate develop antibody production. The type of vascular access may have a role as suggested by the incidence of a higher prevalence of aPL in patients who use a AV graft (22%) vs. AV fistula (6%); even further, vascular access failure was increased significatively in patients with AV grafts and higher aPL titers (74). The presence of these antibodies has not been associated with demographic features, length of time on dialysis, sex, drugs, or chronic B and C hepatitis.

aPL generated have been found to be β2-gycoprotein-1 independent, and their clinical relevance are still unclear (75).

Some authors have not found a relevant clinical relationship or the pathogenic role of antibodies in ESRD (70, 76), but others have found a worse outcome and prognosis in patients with positive aPL (77–79).

Patients with antiphospholipid antibodies that undergo renal transplantation are at risk of thrombosis at any site and graft failure (79–81). McIntyre et al. reported that transplant patients that had positive aPL before the transplant presented a higher rate of graft failure (79). When compared patients who had an early kidney graft failure vs. patients with functioning grafts, the number of patients who had positive aPL were more prevalent in the graft failure group. The histopathologic pattern in patients with APS and graft failure is characterized by thrombotic features and graft infarction (82, 83). Treatment with anticoagulation is not completely preventive for graft loss (79). One report presented good outcomes in patients receiving a renal graft using preoperative immunosuppressive therapy and anticoagulation (84). Therapy with mTOR inhibitors can also be an option that can be used in this group of patients (85).

## Pathophysiology of APS

Clinical studies have shown a strong association of aPL with thrombosis and obstetric morbidity.

### Thrombotic APS

The mechanisms by which aPL cause thrombosis are not completely understood. The underlying pathogenic mechanisms came from animal studies demonstrating that aPL activate endothelial cells, platelets, monocytes, neutrophils, fibroblasts, and throphoblasts. Cellular activation is key in thrombotic APS.

#### Cellular Activation

In platelets, aPL induce expression of thromboxane B2 and fibrinogen receptor glycoprotein IIb/IIIa, resulting in platelet aggregation (86).

In APS there are signs of endothelial activation. aPL can activate endothelial cells to express tissue factor (TF) and adhesion molecules (87, 88). A possible surrogate for endotelial activation is the finding of endothelium-derived microparticles in the circulation of patients with APS (89).

APS patients have increased monocyte TF expression and increased levels of monocyte-derived microparticles, a possible important source of TF (90). TF is the major initiator of coagulation *in vivo*, thus, may be one of the most important contributors to thrombosis.

Neutrophils have recently received attention in APS as they are activated by aPL and release neutrophil extracelular traps (NETs). NETs are composed of chromatin and antimicrobial proteins coming from neutrophils in response to both inflammation and infection. NETs activate platelets and the coagulation cascade and can serve as scaffolding upon which a thrombus can assemble (91). APS patients have elevated levels of low-density granulocytes, a subpopulation of granulocytes that release NETs in exaggerated fashion (92). Moreover, APS patients have impaired ability to degrade NETs (93).

#### Cell Receptors and Signaling Pathways

It has been demonstrated that cell surface receptors that interact with aPL and/or B2GP1 include annexin A2, ApoER2, and TLRs. The intracelular signaling pathways p38MAPK, and subsequent nuclear translocation and activation of NFkB in endothelial cells and monocytes mediate thrombosis in APS (94). On platelets, the main receptors that bind B2GP1/aB2GP1 complexes that induce activation include ApoER2 and glycoprotein Iba. The main signaling pathway is p38MAPK with minor roles of the ERK-1, ERK-2, and phosphatydlinositol 3-kinase/Akt (95).

Recently, it demonstrated the activation of the mammalian target of rapamycin complex pathway in the vascular endothelium of intrarenal vessels from patients with APSN and in the vessels of autopsy specimens from patients with CAPS (96).

#### Complement Activation

Complement activation has a pathogenic role in thrombotic APS (97). Complement activation amplifies coagulation and inhibits fibrinolysis, through C5a, inducing expression of TF and plasminogen activator inhibitor 1 (98).

#### Coagulation Pathways

aPL affect hemostasis at multiple levels. In addition to cellular activation, upregulation of coagulation and inactivation of fibrinolysis are well known mechanisms of thrombosis in APS. Upregulation of TF (99), resistance to activated protein C (100) and complement activation (98) are important mechanisms in aPL-induced thrombosis.

#### Nitric Oxide

Nitric oxide (NO) is a key signaling molecule for the maintenance of normal vascular funtion. Oxidative stress dysregulate the eNOS system which produces superoxide species contributing to vascular dysfunction. In patients with APS, decreased bioavailable NO and increased oxidative stress have been demostrated (101).

### Obstetric APS

The pathogenesis of obstetric APS remains uncertain. Intraplacental thrombosis was thought to be the main pathogenic mechanism of fetal loss. However, placental thrombosis or infarction was not observed in all the cases. There is evidence that aPL impair trophoblastic invasion and human chorionic gonadotrophin production leading to miscarriages, fetal loss, and placental insufficiency (102). Mechanisms relevant to obstetric complications include activation of the complement system with secondary inflammation (103, 104), defective placentation due to interference of anti-B2GP1 with trophoblasts growth and differentiation, and displacement of annexin A5 by aPL-B2GP1 complexes (105).

## Conclusion

The kidney is a major target organ in APS. APSN occurs in primary, secondary, and CAPS. It is characterized by vascular compromise involving large, medium, and small vessels including capillaries. Because clinical features are diverse and not pathognomonic, all physicians treating APS or related diseases need to be aware of these complications. Early recognition and treatment are essential to prevent a poor outcome. The recommended treatment is anticoagulation and tight blood pressure control. In patients who are difficult to treat refractory disease, IGIV, rituximab, or eculizumab could be considered.

## Author Contributions

AT-C, JPH-F, and M-CA contributed to the design of this review. AT-C wrote the first draft of the manuscript. AT-C, JPH-F, M-CA wrote sections of the manuscript. All authors contributed to manuscript revision and approved the submitted version.

## Conflict of Interest Statement

The authors declared no potential conflicts of interest concerning the research, authorship, and publication of this article.
